# Probing DNA Helicase Kinetics with Temperature‐Controlled Magnetic Tweezers

**DOI:** 10.1002/smll.201402686

**Published:** 2014-11-14

**Authors:** Benjamin Gollnick, Carolina Carrasco, Francesca Zuttion, Neville S. Gilhooly, Mark S. Dillingham, Fernando Moreno‐Herrero

**Affiliations:** ^1^ Centro Nacional de Biotecnología, CSICDarwin 3, Campus de Cantoblanco28049MadridSpain; ^2^ DNA–Protein Interactions UnitSchool of BiochemistryMedical Sciences BuildingUniversity of BristolUniversity WalkBristolBS8 1TDUK

**Keywords:** activation energy, AddAB helicase‐nuclease, magnetic tweezers, single‐molecule studies, temperature control

## Abstract

Motor protein functions like adenosine triphosphate (ATP) hydrolysis or translocation along molecular substrates take place at nanometric scales and consequently depend on the amount of available thermal energy. The associated rates can hence be investigated by actively varying the temperature conditions. In this article, a thermally controlled magnetic tweezers (MT) system for single‐molecule experiments at up to 40 °C is presented. Its compact thermostat module yields a precision of 0.1 °C and can in principle be tailored to any other surface‐coupled microscopy technique, such as tethered particle motion (TPM), nanopore‐based sensing of biomolecules, or super‐resolution fluorescence imaging. The instrument is used to examine the temperature dependence of translocation along double‐stranded (ds)DNA by individual copies of the protein complex AddAB, a helicase‐nuclease motor involved in dsDNA break repair. Despite moderately lower mean velocities measured at sub‐saturating ATP concentrations, almost identical estimates of the enzymatic reaction barrier (around 21–24 *k*_B_ *T*) are obtained by comparing results from MT and stopped‐flow bulk assays. Single‐molecule rates approach ensemble values at optimized chemical energy conditions near the motor, which can withstand opposing loads of up to 14 piconewtons (pN). Having proven its reliability, the temperature‐controlled MT described herein will eventually represent a routinely applied method within the toolbox for nano‐biotechnology.

## 1 Introduction

Single‐molecule studies in solution explore phenomena that are driven by energies on the order of *k*_B_ *T* (≈4 piconewton (pN) × nanometer (nm) at *T* = 25 °C = 298 K, *k*_B_ being the Boltzmann constant) and for that reason show a distinct sensitivity to temperature fluctuations within the surrounding medium.^1^ Measuring kinetic parameters of individual proteins or nucleic acids thus requires both thermal accuracy and stability inside the sample cell – quantitative results will otherwise get biased.^2^ Many different approaches have tackled the challenge of efficiently managing the temperature during single‐molecule experiments: either locally via laser heating^3^ or micro‐/nanofabrication,^4^ or macroscopically by enclosing (parts of) the experimental setup^5^ or warming and/or cooling components that are in close thermal contact with the sample.[[qv: 3b]],^6^ For assays based on high–numerical aperture oil‐immersion microscope objectives, macroscopic control is effective and relatively easy to implement;[[qv: 2b]],^7^ indeed, various commercial sample stage and objective temperature controllers exist.^8^

Magnetic tweezers (MT) constitute a surface‐coupled single‐molecule technique that commonly relies on a customized inverted microscope.[[qv: 1b]] In one of its simplest forms,^9^ such a setup contains a pair of permanent magnets whose dimensions are similar to the fluid chamber width and massive in comparison with the size of the biological sample under study. In order to induce a high upwards‐directed force on microspheres tethered to the bottom surface via DNA molecules, the magnet pair needs to approach the chamber from above down to very short distances.^10^ As a result, maximizing the force requires the spacer and upper cover of the fluid chamber to be as thin as possible. At the same time, for accurate tracking of axial microsphere positions, in recent implementations^11^ the objective usually sits on a piezoelectric positioning device that restricts the available space nearby. Consequently, measuring in a MT microscope at different thermal conditions and high forces requires temperature management components that fit into the limited room on both sides of the sample stage and around the objective. In addition, for a proper calibration the fluid chamber configuration must allow for the possibility to probe the thermal conditions near the location of the experiment itself.

Even when only heating above room temperature is considered, meeting these demands with macroscopic temperature control methods proves to be non‐trivial and often involves custom‐built solutions. For instance, in one study a resistive microscope slide was used to heat the fluid chamber of a MT setup from above,^12^ which limited the maximum applicable force. Likewise, in another work, warming of the MT sample cell was achieved by placing thin heating elements on top of it, besides adjustments of the ambient temperature in the laboratory.^13^ A commercial MT apparatus with thermally stabilized sample stage^14^ has been employed for monitoring enzyme activity at different temperatures^15^ but – to the best of our knowledge – lacks an accurate measurement of the temperature in the interior of the cell.

Here, we propose a simple way to control the thermal conditions inside the fluid chamber of a customized MT apparatus without the mentioned caveats. We adjust the temperatures of sample cell holder and oil‐immersion objective at the same time to reduce thermal gradients throughout the buffer volume, as recommended. However, our approach based on flexible heating elements and small high‐precision temperature sensors needs minimum extra space, therefore preserving the range of measurable forces and eluding fundamental changes in the experimental setup. Moreover, it can be adapted not only to related MT systems, but also to other inverted microscope–based methods that may require access to the area on top of the fluid chamber, e.g. total internal reflection fluorescence (TIRF) microscopy[[qv: 4c]],^16^ or ionic current sensing combined with optical tweezers.^17^

We tested the efficiency of the temperature‐controlled MT via a recently described single‐molecule assay with the adenosine triphosphate (ATP)‐dependent helicase–nuclease AddAB from *Bacillus subtilis* 18 – a protein exhibiting stable unwinding and infrequent nicking action, responsible for initial DNA end resection during double‐stranded (ds)DNA break repair by homologous recombination.^19^ In order to gain deeper knowledge about the kinetic parameters governing the coupled unwinding and translocation reactions of this enzymatic model system, we compared results from MT experiments at several temperatures with data obtained from stopped‐flow ensemble measurements using related DNA substrates. We expected both techniques to yield comparable temperature dependences of AddAB helicase activity, represented by similar activation energy barriers describing the same molecular process.

## 2 Results

### 2.1 A Versatile Temperature Control Method for MT‐type Microscopes

**Figure 1:**
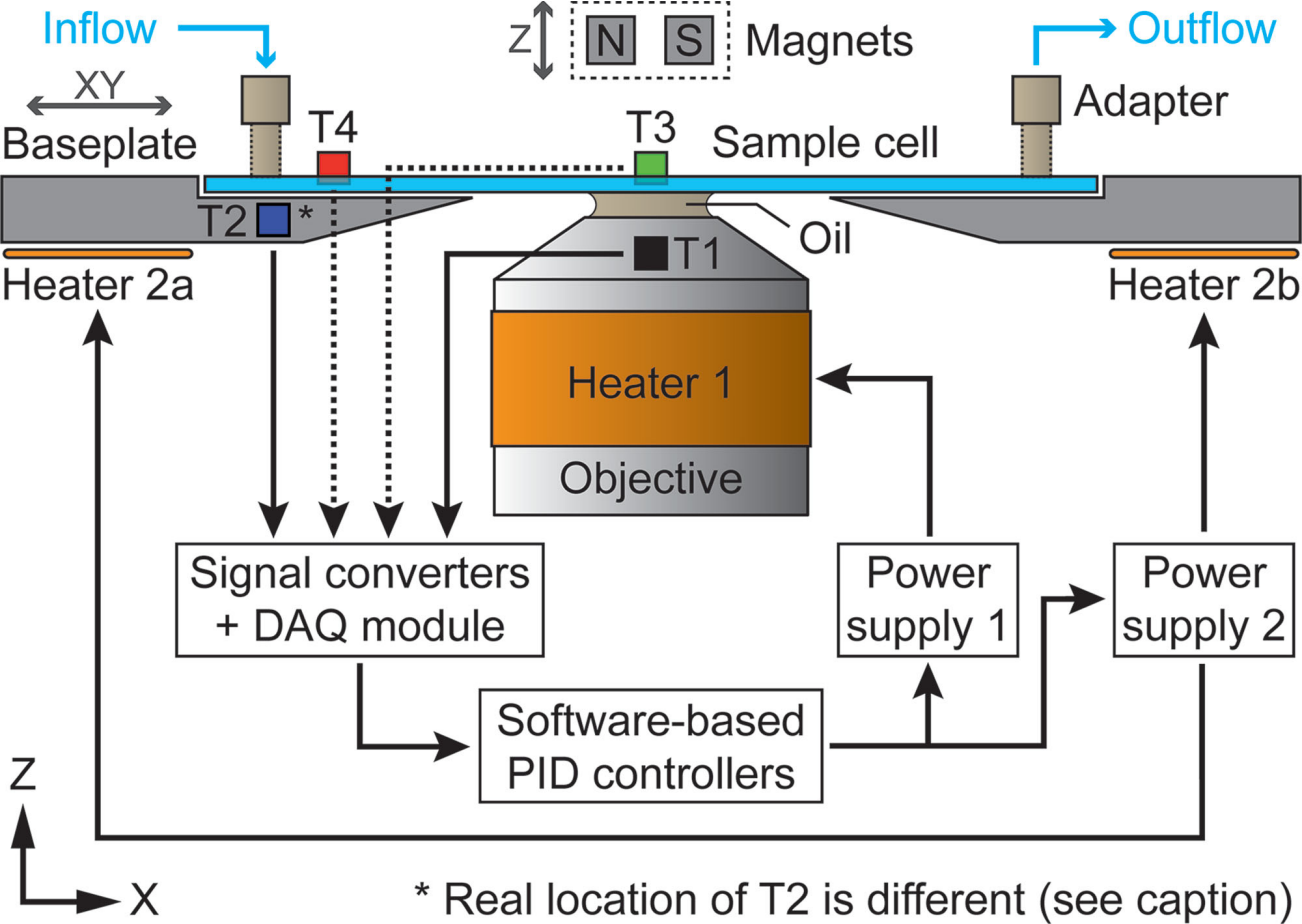
Schematic representation of the thermally controlled magnetic tweezers (MT) apparatus. The setup (side view, not to scale) contains one resistive heating foil (Heater 1) around the oil‐immersion microscope objective (fixed in XY) and two identical heating foils (Heaters 2a/b) connected in series underneath the sample holder baseplate (moveable in XY). Sample cell, baseplate and Heaters 2a/b are represented in a cross‐sectional view. The space on top of the sample cell (only microfluidic adapters shown) is left unchanged so that a pair of permanent magnets (moveable in Z) can be brought close to the upper glass surface. Temperatures are measured by up to four platinum resistance (Pt100) thermometers (T1–T4) and converted into voltages afterwards. Sensors T1 (black) and T2 (blue) are permanently fixed and provide the values from objective and baseplate used for the temperature control feedback. The real location of T2 is halfway between Heaters 2a/b in X and offset in Y (behind the objective in this representation, see Figure S1). Sensors T3 (green) and T4 (red) are optionally attached to measure the buffer temperature *inside* a modified fluid chamber near the lower glass surface (see Figure S2). The stabilizing feedback loop relies on a data acquisition (DAQ) module, two software‐based proportional‐integral‐derivative (PID) controllers and two programmable power supply units, one per heater circuit (see Experimental Section).

**Figure 2:**
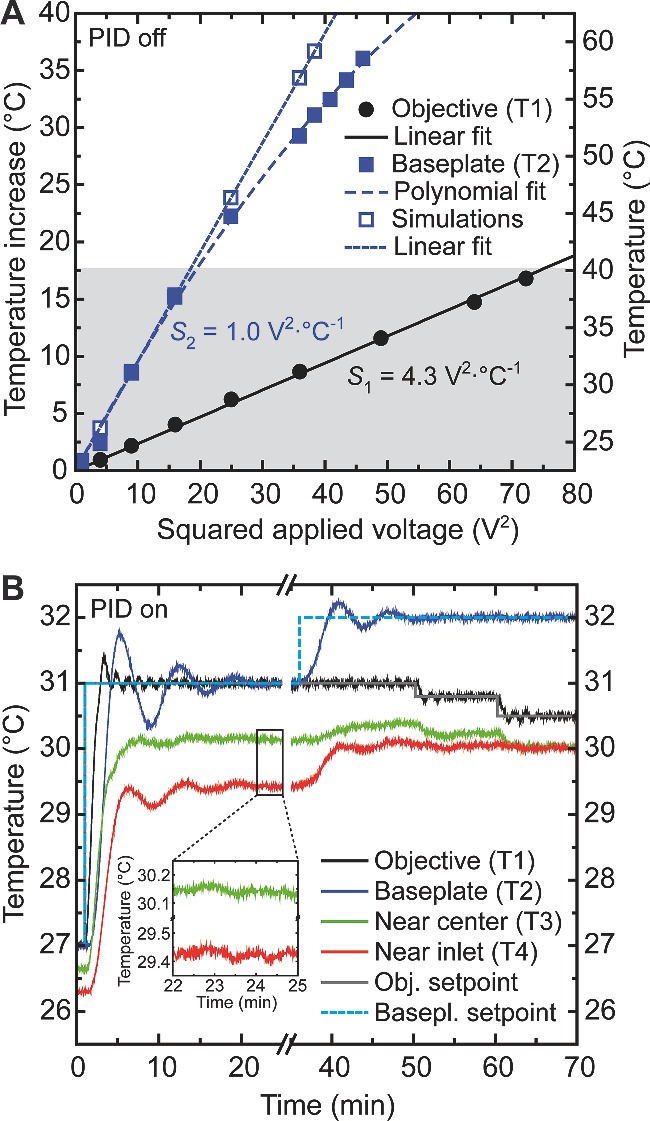
Assessment of heating parameters and PID control performance. (A) Sensitivities of objective (black: Heater 1 ↔ T1) and baseplate (blue: Heaters 2a/b ↔ T2) circuit determined by independent measurements of stabilized temperatures for different power supply voltages *without PID feedback*. A temperature increase ▵*T* = 0 corresponds to ambient conditions (*T*_ambient_ ≥ 23 °C). (B) *Before axis break*: the time response *with PID feedback* after a typical setpoint change (27 → 31 °C), monitored using all four Pt100 sensors (at 2 Hz). The temperature inside the sample cell (no buffer flow) stabilizes to ±0.1 °C of precision or better after 20 minutes (see inset) and can be held for whatever time is necessary. *After axis break*: appropriate combinations of objective (grey) and baseplate (cyan) setpoints can yield equivalent temperatures near center (T3, green) and inlet (T4, red) of the sample cell.

**Figure 3:**
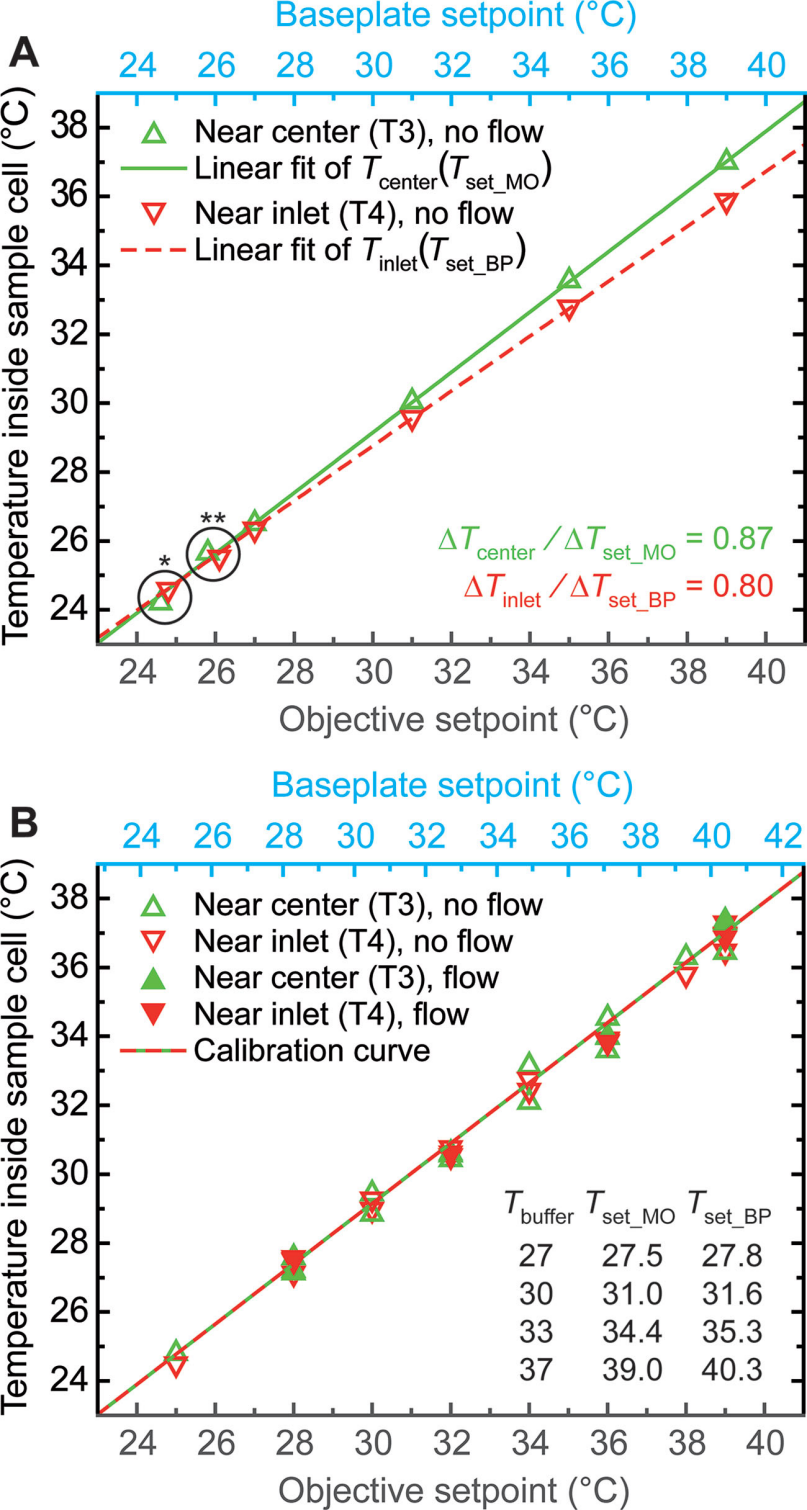
Calibration of the temperature inside the sample cell. (A) Distinct linear temperature increments probed inside the fluid chamber (no buffer flow) near center (*T*_center_, green triangles and fit) and inlet (*T*_inlet_, red triangles and fit) by applying *equal temperature setpoints* to objective (*T*_set_MO_, grey) and baseplate (*T*_set_BP_, cyan). Encircled data points correspond to ambient measurements (no heating) before (*) and after (**) assessment of the temperature values for 27 °C ≤ *T*_set_MO_ ≡ *T*_set_BP_ ≤ 39 °C. (B) The linear fits in (A) taken as references (see Results) yield an optimum relation between objective and baseplate setpoints for the condition *T*_center_ − *T*_inlet_ ≡ 0 (note the now *unequal setpoint axis scales*): all temperature measurements near center and inlet – both with the buffer at rest (empty triangles) and with an overall buffer flow velocity of about 1 μL·s^−1^ (filled triangles) – collapse on a straight line (green/red, valid for *T*_ambient_ ≥ 23 °C) matching both fits in (A). A corresponding table with setpoint values necessary to achieve characteristic temperatures *T*_buffer_ for MT experiments is shown as an inset.

We broadened the capabilities of a widely used MT setup by implementing a thermal control assembly (see **Figure** **1** and Supporting Information) for single‐molecule experiments between room temperature and 40 °C (see **Figure** **2**). To maintain the entire functionality of the customized inverted microscope, we combined thin heating foils and resistive temperature detectors at the objective and underneath the sample holder baseplate, yielding equivalent results in two complementary MT configurations (see Experimental Section). Using a modified fluid chamber with extra apertures in the top cover glass, we could integrate two additional thermometers for probing the buffer temperature directly and in this way calibrate a homogeneous temperature profile throughout the sample volume (see **Figure** **3**).

### 2.2 Performance of the Heating/Heat‐Sensing Elements

#### 2.2.1 Feasible Measurement Range

The thin‐foil heater circuits showed ohmic behavior up to ∼40 °C (grey‐shaded area in Figure 2A): the observed temperatures increased linearly with the heating power *P*_heat_ = *V* × *I* = *V* ^2^/*R* (*V* being the applied voltage, *I* the measured current and *R* the resistance). Above 40 °C, Heater 2a/b data presented an apparent departure from Joule heating and adjusted to a polynomial function rather than to the straight line obtained from computer simulations of the baseplate alone (see Figure 2A). In any case, when used simultaneously, the setpoints of both temperature control circuits never exceeded 43 °C.

#### 2.2.2 Temperature Stability

After optimization of the proportional (P), integral (I) and derivative (D) gains (see Experimental Section), the system achieved at least 0.1 °C of precision – a value that could be maintained for days if required – within less than half an hour upon a considerable temperature setpoint change (see inset of Figure 2B). Despite a slower response and larger initial overshoots, baseplate temperatures always stabilized to the noise level of objective temperatures within the same amount of time (see Figure 2B, before axis break). Thermometers T3 and T4 (in contact with the buffer solution) depicted values that were in general lower than those of T1 and T2 and normally unlike, but which could be balanced by proper setpoint adjustments. Besides, the heating circuits showed little cross‐talk, i.e. objective heating mainly influenced sensor T3 and baseplate heating mainly sensor T4 – provided the setpoints were close to each other (see Figure 2B, after axis break).

### 2.3 Calibration of the Thermal Conditions for Single‐Molecule Experiments

For an experimental configuration as in Figure 1 and equal setpoints *T*_set_, the two heat transfer processes – (i) from the objective (MO) via the immersion oil drop towards the sample cell center and (ii) from the baseplate (BP) towards the sample cell edges (near buffer in‐/outlet) – satisfied slightly differing linear fits defined by the following representative equations: *T*_center_ = 0.875 × *T*_set_MO_ + 2.910 °C and *T*_inlet_ = 0.796 × *T*_set_BP_ + 4.870 °C. The two straight lines intersect at a temperature of about 25 °C, close to the mean room temperature during these measurements (see Figure 3A). To minimize any gradient across the long direction (X) of the fluid chamber, we imposed the condition *T*_center_ ≡ *T*_inlet_ ≡ *T*_buffer_ and raised *T*_set_BP_ relative to *T*_set_MO_ accordingly. Measurements of *T*_center_ and *T*_inlet_ corresponding to the same combinations of *T*_set_MO_ and *T*_set_BP_ = 1.098 × *T*_set_MO_ − 2.461 °C, but performed on various days or with different sample cells, resulted in equivalent temperatures inside the fluid chamber with a typical repeatability of ±0.5 °C (see Figure 3B).

### 2.4 Temperature Dependence of DNA Translocation by a Helicase–nuclease Prototype

**Figure 4:**
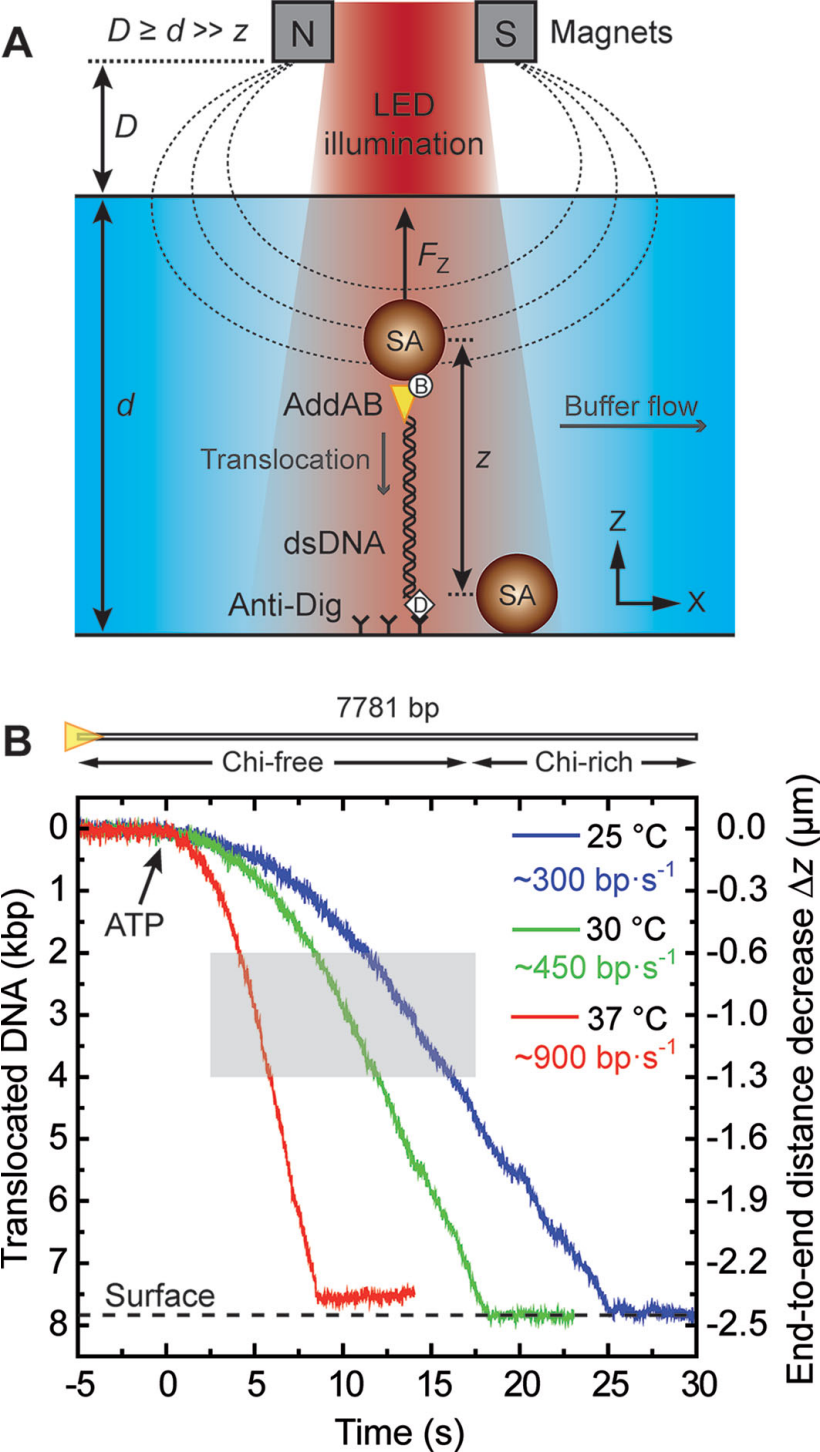
DNA translocation by the model helicase‐nuclease AddAB. (A) Schematic view of the experimental layout for MT experiments (not to scale). A pair of permanent magnets at a distance *D* above the sample cell (thickness *d*) exerts a constant pulling force *F*_Z_ on a magnetic sphere in solution that stretches a DNA–protein construct to an initial end‐to‐end distance *z*. Note that, for *F*_Z_ = 3 piconewtons (pN) – in MT_1_‐configuration with standard/thick sample cells (see Experimental Section) – *D* ≈ 500 μm > *d* ≈ 200 μm >> *z* ≈ 2.4 μm < *L*_0_ ≈ 2.6 μm (DNA contour length). Flushing buffer with adenosine triphosphate (ATP) initiates translocation. (B) Three example traces (3 pN, 1 mM ATP) showing single‐molecule activity of AddAB along a 7.8 kilo–base pair (kbp) DNA substrate devoid of regulatory crossover hotspot instigator (Chi) sites within the first 5 kbp, measured with our thermally controlled MT at 60 Hz (MT_1_, standard/thick cells). Time *t*_0_ ≈ 0 denotes the apparent arrival of ATP at the enzyme. Due to variations in the attachment point of AddAB at the magnetic sphere, not all (temperature‐corrected) translocation traces span the complete end‐to‐end distance. The grey rectangle indicates the substrate region (2–4 kbp) considered for statistical analysis of instantaneous translocation rates (see Figure 6).

**Figure 5:**
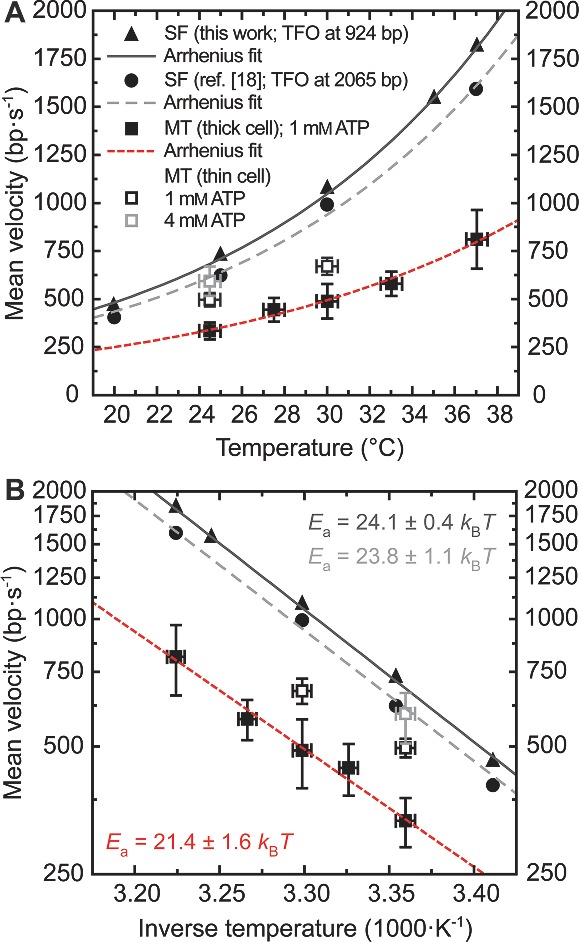
Mean AddAB translocation rates as functions of buffer temperature. (A) Single‐molecule MT data acquired at 3 pN (squares) are compared with results from stopped‐flow (SF) ensemble measurements (triangles: this work; circles: data from Reference 18 that rely on the displacement of a triplex‐forming oligonucleotide (TFO, see Experimental Section and Figure S4). SF and MT data sets (filled symbols) obtained with 1 mM of ATP in the reaction buffer – the latter with standard/thick sample cells – are fitted by single exponential functions according to the Arrhenius relation (Equation (1)). Empty symbols indicate MT experiments with thin cells (*d* ≈ 100 μm in Figure 4A) at the same (black) and a fourfold higher (grey) volumetric ATP concentration, which give rise to 30–50 and ∼85% faster rates, respectively, practically recovering the bulk values in the second case. For all single‐molecule measurements, error bars along the X‐axis correspond to the accuracy in temperature (±0.5 °C) as estimated from the typical spread observed among independent measurements in Figure 3B. Error bars in Y represent the standard deviation (SD) of at least 23 (3) data points at each temperature acquired in thick (thin) fluid chambers, with the standard error of the mean (SEM) being always smaller than or equal to the symbol size. For the ensemble measurements, uncertainties in temperature and velocity correspond to the symbol size. (B) Arrhenius plots of the results presented in (A): the exponential fits now show as straight lines with systematic offset but similar slopes, representing comparable activation energies *E*_a_ ≈ 21–24 *k*_B_ *T*. Uncertainties of *E*_a_ correspond to the standard errors (SEs) returned from the fits; the remaining error bars are as in (A).

We applied the MT microscope with thermal control unit to investigate the single‐molecule activity of the *B. subtilis* AddAB protein complex at various temperatures (**Figure** **4**A). Translocation traces taken between 24 and 37 °C at 3 pN of load applied on the protein presented common features: an onset phase due to ATP influx and occasional slowdowns at characteristic positions – beyond the initial 5 kilobase pairs (kbp) – of the DNA substrate (see Figure 4B). Raising the temperature from ambient to physiological conditions increased the mean translocation velocity *v*_MT_ about threefold. An exponential fit defined by the general Arrhenius relationship

(1) $$v(T)=v_{0}\times e^{-E_{a}/\left(k_{B}T\right)}$$

(where *v*_0_ is the hypothetical rate at infinite temperature, *E*_a_ the activation energy barrier of coupled unwinding and translocation, and *k*_B_ *T* the thermal energy of the surrounding heat bath) yielded a temperature coefficient *Q*_10_ ≡ *v*_MT_(*T* + 10 °C)/*v*_MT_(*T*) ≈ 2. This parameter remained valid for the equivalent fits of translocation rates *v*_SF_ obtained in bulk from two different stopped‐flow (SF) fluorimetry data sets (see **Figure** **5**A). Re‐plotting the dependencies in Arrhenius representation shed light on (i) a systematic difference corresponding to *v*_MT_thick_(*T*) ≈*v*_SF_(*T*)/2 when comparing the *average* single‐molecule and bulk velocities obtained under equal volumetric ATP conditions and with standard/thick MT sample cells (filled symbols in Figure 5, see Experimental Section); and – at the same time – (ii) activation energy constants of 21 ± 2 and 24 ± 1 *k*_B_ *T* (equivalent to values around 52 and 59 kJ·mol^−1^, or 12 and 14 kcal·mol^−1^), respectively, which were similar within experimental error (see Figure 5B).

**Figure 6:**
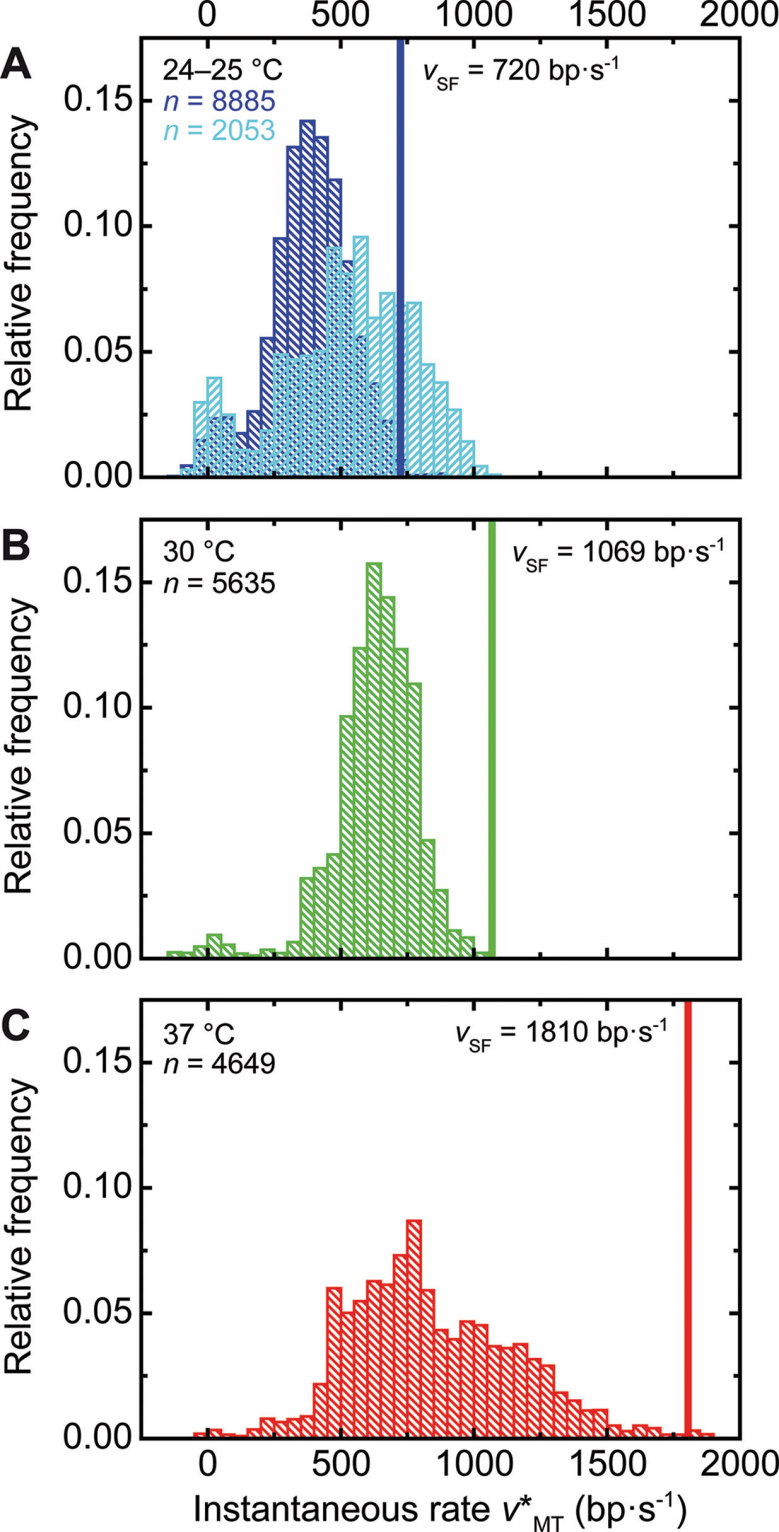
Instantaneous AddAB velocities according to MT data at 3 pN. Considering the region between 2 and 4 kbp from the proximal end of the 7.8 kbp DNA substrate used in our single‐molecule assays (grey rectangle in Figure 4B), instantaneous rates v*_MT_ derived from translocation traces (*n* = total number of respective rate values) at (A) 24–25, (B) 30 and (C) 37 °C are accumulated in histograms (dark blue, green and red: thick sample cells, 1 mM ATP; light blue: thin sample cells, 4 mM ATP). Disorder exhibited by the translocating proteins determines the broadened shape of the resulting distributions.^18^ Random pauses corresponding to rate values close to zero mainly occur at lower temperatures. Mean velocities *v*_SF_ from stopped‐flow triplex displacement experiments (inferred from the data shown in Figure S4 and depicted in Figure 5) are represented by straight vertical lines and located close to the highest instantaneous rates observed in thick fluid chambers. The use of thin chambers and higher ATP concentrations shifts/expands the histogram in (A) to larger speeds, with its mean value approaching the bulk rate.

Using thinner MT cells while keeping the buffer volume flow velocity unchanged, mean translocation rates (empty symbols in Figure 5) came closer to stopped‐flow results and resembled them at room temperature and higher ATP concentration (4 mm). Accordingly, a more detailed analysis of the *instantaneous* single‐molecule rates v*_MT_(*T*) corresponding to a 2 kbp–long section of all translocation traces (obtained at 3 pN) revealed an overlap of *v*_SF_(*T*) with the high‐velocity tails of the v*_MT_(*T*)‐distributions. The distribution center shifted towards the mean bulk rate for the data obtained with thin cells at ambient conditions and high amounts of ATP (see **Figure** **6**).

## 3 Discussion

### 3.1 Relevance of Our Temperature Control Strategy

We have formulated a general scheme for single‐molecule experiments at physiological temperatures with permanent magnet–based MT setups. Our macroscopic control method combines thin‐film heating elements with small thermometers and involves a simple procedure to calibrate the temperature inside the fluid chamber. This constitutes a straightforward and space‐saving option for users of vertical MT instruments – or, in general, of experimental setups that rely on customized inverted microscopes – who wish to vary the thermal conditions when studying the mechano‐dynamics of (biologically significant) objects at the nano‐/micrometer scale.

Other types of surface‐coupled single‐molecule instrumentation may favor alternative realizations of macroscopic thermal control. For instance, in a transverse MT apparatus a Peltier chip was used to adjust the measurement temperature,^20^ whereas a combination of resistive heating foils and Peltier elements was needed for managing the conditions in a dual‐beam optical tweezers instrument from both sides.^21^ When compared with our system, both designs yield similar values of thermal precision (∼0.1 °C) and accuracy (∼0.5 °C) but require a completely different layout or at least substantial modifications of the sample cell holder parts that would be hard to put into practice in axial (vertical) MT setups.

Recently, it turned out that a single commercial objective heater can be sufficient for MT measurements at high temperatures with the buffer at rest.^22^ Testing the performance of our temperature control assembly without warming the baseplate, we found that at 37 °C and a constant buffer flow of ∼1 μL·s^−1^ through standard/thick (200 μm) sample cells, the mean single‐molecule translocation rate of AddAB dropped by ∼10% (data not shown). This decrease should be more pronounced for higher flow velocities, which may however cause experimental problems due to more frequent video tracking errors and detachments of DNA tethers. The added complexity of sample cell heating is paid off by further advantages though: (i) because of the more homogeneous thermal profile within the fluid chamber, convection currents are minimized, a fact that may be essential when acquiring long time series of data, e.g. during measurements of DNA looping dynamics^23^ or particle diffusion in confined geometries;^24^ and (ii) in order to reach a certain buffer temperature, less power needs to be applied at the objective itself, which reduces thermal stress and should increase the lifetime of the optical components.

In brief, we are confident to anticipate that for experiments above ambient conditions with good thermal accuracy over extended periods of time, our versatile way of implementing temperature control in MT applies as well to other (single‐molecule) instrumentation with little space available near the fluid chamber – especially to configurations that require continuous buffer flow as in chambers used with multiple laminar streams.^25^ Assemblies capable of detecting fluorescence can additionally supply non‐contact temperature information via thermally induced fluorophore quenching.^26^

### 3.2 Complementary Temperature Assessment

To estimate the thermal conditions at the bottom surface inside the sample cell, we (i) monitored temperature‐induced shape changes of different waxes with well‐defined melting points as previously described,^27^ and (ii) performed finite‐element simulations of the symmetrically warmed baseplate (data not shown). Both methods confirmed the expected thermal characteristics up to ∼40 °C. The (experimentally determined) nonlinear response of the Heater 2a/b circuit – significant for temperatures ≥45 °C not addressed during MT experiments (see Figure 2A) – originates most likely from increased heat dissipation to the surroundings. For instance, even though the baseplate is physically disconnected from other (metallic) microscope components, its bottom surface with the attached heating elements (see Figure S2B) floats only ∼1 mm above a large aluminum breadboard that likely contributes to the effect (an additional layer of thermally insulating material would be difficult to implement due to space restrictions). Future simulations with improved boundary conditions should take such circumstances into account to provide a more realistic picture of the temperature distribution throughout the cell.

### 3.3 Time Response and Measurement Range of the Thermostat

We are aware of the fact that our macroscopic control technique requires relatively long stabilization times, in particular when lowering a previously raised setpoint (see Figure S3). Note that the objective – aside from the baseplate (see above) – is *not* thermally isolated from the rest of the microscope. Without any active cooling mechanism, the use of e.g. plastic spacers may reduce temperature fluctuations,[[qv: 2b]] but normally leads to additional, undesired delays during relaxation.^28^ Apart from that, due to inevitable signal oscillations after a significant setpoint change (see Figure 2B), our system is most useful for measurements at constant temperatures. To partly alleviate these issues, one might place the whole apparatus in a refrigerated environment (not applicable in our case) or use a better heat conductor for the baseplate. We chose stainless steel as the preferred compound because despite its only modest thermal conductivity of about 15–20 W·m^−1^·K^−1^ it exhibits superior chemical resistance and mechanical stability when compared to e.g. aluminum.

The upper temperature limit (∼40 °C) inside the fluid chamber derives from the maximum value that we considered for safe operation of our oil‐immersion objective (43 °C) after having consulted the manufacturer and taking into account initial temperature overshoots. Higher values are possible,^22^ but (long‐term) detrimental effects on the equipment can no longer be excluded.^8^ The lower boundary is given by the temperature in the laboratory (kept constant at 24.5 ± 1.5 °C), resulting in a measurement interval sufficient for studying the activity of proteins from most mesophilic pro‐ and eukaryotes. In experiments that necessitate a broader temperature range, values below ambient conditions, fast temperature changes or defined gradients, other strategies of thermal control may be preferred (see Introduction). However, one key advantage of MT assays addressing large areas of the fluid chamber surface is that they are highly parallelizable,^29^ which favors macroscopic temperature management. This also implies that, even if we could actively lower the temperature of our setup and accelerate its step response, the time needed to reach stability in single‐molecule assays would still be determined by thermal equilibration within the relevant chamber regions – achieved when no more significant drift is observed on the camera image.

### 3.4 How Reliable are Our Calibrations?

Optimally, one should keep track of the temperature inside the sample cell at all times. In our case, attaching thermometers as shown in Figure 1 while performing MT experiments would however compromise the optical image essential for accurate position detection. Conversely, sideward introduction of an appropriate sensor into the fluid chamber would require further changes to the holder parts, potentially affect the chamber thickness, and ultimately make sample preparation more complicated – thus reducing the throughput of experiments. Fortunately, due to the small channel height of our sample cells (100–200 μm, see Figure 4A), we can assume that single‐molecule measurements conducted a few micrometers above the lower coverslip surface are subject to the thermal settings calibrated beforehand. Still, the results presented in Figure 3 are only accurate for a specific configuration of the inverted microscope: the setpoints to achieve a certain temperature inside the fluid chamber depend on the thermal profiles of objective and sample cell holder (equivalent for our two MT configurations, see Experimental Section), apart from the exact XY‐positions of thermometers T3 and T4 relative to the immersion oil drop. After a substantial change of any of these parameters the temperature calibration procedure should therefore be repeated. On this account, the confidence intervals of ±0.5 °C extracted from Figure 3B and depicted in Figure 5 do include uncertainties due to (i) changes in the XY‐position of the sample stage relative to the objective during consecutive experiments with different fluid chambers and (ii) variations in the experimental conditions when comparing data acquired on various days. In summary, it is safe to say that our system works properly without direct monitoring of the buffer temperature during single‐molecule experiments.

### 3.5 Comparison of MT and Stop‐Flow Translocation Rates

We have shown that the temperature‐controlled MT microscope serves for studying enzyme kinetics between 24 and 37 °C and have compared single‐molecule results with their counterparts from ensemble measurements. As observed in previous MT experiments with the model helicase–nuclease AddAB, occasional pauses around 5.5 kbp from the proximal end of the DNA (see Figure 4B) correlate with substrate regions containing individual crossover hotspot instigator (Chi) sequences in the correct orientation.^18^

We first supposed that the systematic difference between initially determined single‐molecule and bulk translocation rates (filled symbols in Figure 5) might stem from two features of the MT assay that are not present in stopped‐flow experiments: (i) a thousandfold larger microsphere directly attached to the biotinylated AddAB complex and (ii) a constant upwards‐directed force applied by the magnets (see Figure 4A). However, these aspects have proven to play only a minor role. Concerning the first point, in a previous MT study the use of an alternative version of AddAB – containing a longer linker between protein motor and biotin tag peptide sequence – gave rise to identical results in control experiments,^18^ thus excluding a steric hindrance effect of the microsphere on the motor. In addition, AddAB advancing at a speed of ∼1 kbp·s^−1^ ≈ 0.3 μm·s^−1^ feels a negligible drag force induced by the sphere (∼0.003 pN), and the lateral drag caused by the laminar flow of buffer (see below) adds no significant contribution either. With respect to the second argument, Figure S5 illustrates that a magnetic pulling force of 3 pN pointing opposite to the direction of translocation has little restraining impact on the protein: increasing the load on AddAB up to 14 pN hardly reduces its pause‐free unwinding rate. This observation is striking, yet in accordance with a previous single‐molecule study showing forward motion of the closely related *Escherichia coli* RecBCD helicase‐nuclease^30^ – which performs the same net reaction as AddAB – at non‐saturating ATP concentrations against loads of up to 8 pN applied on the protein.^31^ Theoretically, nucleic acid motors that exploit the energy derived from nucleoside triphosphate hydrolysis are able to generate forces up to the DNA overstretching transition (and beyond), depending on their step size.^32^ It therefore comes as no surprise that protein assemblies (like AddAB/RecBCD) that function according to a Superfamily‐1 helicase mechanism can exhibit sufficiently high strengths to remove obstacles along their way.^33^

Based on the results of MT measurements at 3 pN with thin (100 μm) sample cells (empty squares in Figure 5), we propose that the moderate divergence observed between bulk and single‐molecule rates of helicase activity mainly arises from reduced ATP concentrations near the enzyme in our MT assays. Note that this effect is independent of other technical modifications: control experiments using the original (standard/thick) cells at 3 pN generated with various magnet alignments gave identical results for AddAB (data not shown). While stopped‐flow devices provide a defined reaction volume with homogeneous chemical energy distribution after practically instantaneous, turbulent mixing of two reservoirs, (surface‐coupled) single‐molecule techniques that rely on a *single* stream of buffer depend on much slower, laminar phenomena for reaction initiation: shrinking the MT fluid chamber height by 50%, the parabolic flow profile in microfluidic channels^25^ implies a fourfold increase of the linear flow velocity *v*_flow_(*z*) in X at the initial distance *z* ≈ 2 μm of the protein from the coverslip (see Figure 4) – but *v*_flow_(2 μm) still remains below 5 (10)% of the maximum (average) velocity along the channel centerline (see Supplementary Experimental Section). This suggests that during the course of a translocation run (∼10–20 s, see Figure 4B), while injecting reaction buffer with ATP – and depending on the interplay between average flow rate and sample cell geometry –, due to the gradual concentration rise there may be insufficient time to reach the desired (volumetric) level of chemical energy at the protein, and only a large excess of ATP saturates its vicinity early enough (empty grey square in Figure 5). Along the same lines, the histograms of instantaneous single‐molecule rates – governed by static and dynamic disorder^18^ – reveal that, even though AddAB eventually reaches the average bulk velocities at sub‐saturating conditions, the molecular motor can only maintain (and transiently surpass) them in the case of faster laminar flows and increased amounts of biological fuel (see Figure 6). This reasoning is in agreement with a critical ATP concentration dependence of DNA unwinding observed for the same enzyme in a TIRF microscopy assay^16^ and may provide an additional explanation for previously reported discrepancies between independent single‐molecule translocation studies of RecBCD carried out in different experimental setups at various distances from the sample cell surface.[[qv: 5b]],^30^, ^31^

### 3.6 Temperature Dependence of AddAB Activity

The molecular mechanism by which the AddAB helicase‐nuclease moves along and unwinds the duplex DNA substrate appears to be unaffected by the choice of the technique and potential ATP concentration differences: the Arrhenius fits in Figure 5B represent equivalent activation energies *E*_a_ on the order of 21–24 *k*_B_ *T*. Note that none of the fits are error‐weighted because the scattering of the mean rate values contributes more than the standard error of the mean (SEM) at each temperature.^13^ Consequently, the overall uncertainties of *E*_a_ consist for the most part of the standard errors (SEs) returned after successful curve fitting. These comprise (i) random error sources apart from temperature fluctuations that may play a role in stopped‐flow measurements^13^ and (ii) intrinsic heterogeneities among the set of individually studied molecules that primarily determine the stochastic uncertainty in MT experiments.^18^ Slight alterations of bulk assay methods (see Experimental Section) probably account for the small offset between the two stopped‐flow data sets (triangles/circles in Figure 5). Applying a statistical error weight to the single‐molecule data (filled squares in Figure 5), a fit with smaller residual sum of squares but similar slope (corresponding to 21.1 ± 1.6 *k*_B_ *T*) arises (data not shown). A Gaussian uncertainty propagation for *E*_a_(*v*(*T*),*T*) adds at most another ±0.2 *k*_B_ *T* to the total uncertainty (see Supplementary Experimental Section), proving that the SEs from the fits indeed represent the largest contribution. Taken together, all the information from MT and stopped‐flow assays yields an activation energy for AddAB that falls in the range between 19 and 25 *k*_B_ *T*.

The obtained *E*_a_‐values compare with those reported for other translocating proteins within similar temperature ranges: for example, RecBCD shows temperature‐dependent unwinding defined by an activation energy of 10–19 kcal·mol^−1^ (17–32 *k*_B_ *T*) as inferred from several bulk measurements;^34^ at the single‐molecule level, two separate optical tweezers studies demonstrated that transcription elongation by the *E. coli* RNA polymerase obeys a temperature dependence described by *E*_a_ = 10–13 kcal·mol^−1^ (17–22 *k*_B_ *T*);^35^ and in a MT assay, the activation energy of translocation by the dsDNA translocase *Eco*R124I was shown to be approximately 16 *k*_B_ *T*.^13^ In summary, the apparent energy barrier of coupled dsDNA unwinding and translocation by the AddAB helicase–nuclease determined herein is in agreement with the results for a functional homologue (RecBCD) and slightly higher than the one observed for a pure translocase (*Eco*R124I) that lacks unwinding activity.

As for the mechanistic details governing the increase of AddAB translocation rate with temperature, we may speculate that they correspond to a combination of enhanced ATPase activity and decreased stability of the dsDNA substrate – with the former playing a more prominent role (note that the latter has proven primarily important for ring‐shaped helicase motors classified as “passive”,^36^ which however employ totally different DNA unwinding mechanisms^37^ that are not relevant to heterodimeric AddAB^38^ or other Superfamily 1 helicases.^33^ To deconvolute both effects and check the validity of an ATP coupling efficiency close to 1 ATP·AddAB^−1^·bp^−1^ as recently observed at 37 °C in bulk,^39^ prospective trials should provide estimates of the ATPase rate at lower temperatures and address an independent destabilization of the substrate molecule by altering DNA sequence or buffer salt concentration.

## 4 Conclusions

We have demonstrated the versatility of a customized thermal control unit implemented in vertical MT by measuring the single‐molecule kinetics of a DNA motor protein at various temperature, force and ATP settings. Proof‐of‐principle experiments with the bacterial helicase–nuclease complex AddAB comply with bulk assays and yield apparent activation energies of translocation that match published data for related enzymes. This shows that the thermostated optical microscope described here is well‐suited for precise studies of thermally induced activity changes of (sub)micron‐sized objects. The temperature management device allows for accurate calibration of the thermal conditions inside the sample cell and can be easily adapted to other inverted microscope configurations, which suggests an important benefit for state‐of‐the‐art techniques such as hybrid DNA origami nanopores,^40^ photo‐activated localization microscopy (PALM),^41^ or optical torque wrenches.^42^ We expect the proposed design to find use as a powerful tool for exploring the principles of nucleic acid–protein interactions and other fundamental processes at the interface between biophysics and nanotechnology.

## 5 Experimental Section

*Proteins and DNA Substrates*: Native‐43 and biotinylated‐AddAB^16^ proteins were purified as previously described. DNA molecules for MT experiments were fabricated by polymerase chain reaction (PCR) as stated by Carrasco et al.;^18^ in this work, the 7.8 kbp substrate called Chi10‐Rev‐Rev – which is deficient of regulatory *B. subtilis* Chi (5’‐AGCGG) sites within the first 5 kbp^18^ – was used. Stopped‐flow kinetics were measured using a very similar substrate named pSP73‐JY0‐TFO, which shares the same parent plasmid as Chi10‐Rev‐Rev and also lacks Chi sequences within the relevant substrate region. For details of construction of the parent plasmid pSP73‐JY0, consult the supplementary data of Reference^16^; the plasmid pSP73‐JY0‐TFO additionally contains an engineered triplex binding site at a BstBI site, which is 926 bp away from an XbaI site. To constitute the substrate DNA for stopped‐flow measurements as described before,^39^ the purified plasmid was linearized with XbaI and annealed to a 22 nucleotides (nt)–measuring triplex‐forming oligonucleotide (TFO, 5′‐TTCTTTTCTTTCTTCTTTCTTT) that was fluorescently 5′‐labelled with tetramethylrhodamine (TAMRA).

*MT Instrument*: Our setup is based on the design first described by Strick et al.[[qv: 9a]] and similar to the one illustrated in Reference 13. We use two configurations (MT_1_ and MT_2_) whose differences do not affect temperature calibration inside the fluid chamber (MT_2_ serves for high‐force measurements). Both versions rely on (i) a pair of cubic rare‐earth magnets in horizontal/vertical alignment,^10^ (ii) a high‐numerical‐aperture objective and (iii) a charge‐coupled device (CCD) or complementary metal–oxide semiconductor (CMOS) camera for video‐based position detection under bright‐field light‐emitting diode (LED) illumination (see Table S1 for details). In both cases, pulling forces in Z were calibrated with 5–10% precision at ambient conditions according to a well‐established protocol.^9^ Note that the obtained values are invariant to temperature changes.^20^ To accommodate two heating elements (Heaters 2a/b) and one permanent temperature sensor (T2) to the sample cell holder (see Figure 1), an appropriate stainless‐steel baseplate was designed and fabricated (see Figure S1) – no other changes were introduced with respect to the setup used in Reference 18 (MT_1_). Fluid chambers constituted a sandwich of two #1 coverslips (BB024060A1, Menzel‐Gläser), with either two (*standard/thick*: sample cells with inner height of 200 μm, for temperature calibrations and single‐molecule experiments at 3 pN in MT_1/2_) or just one (*thin*: sample cells with inner height of 100 μm, for single‐molecule experiments at 3–14 pN in MT_2_, see Figure S5) layer of plastic paraffin film (Parafilm M, Bemis) in between, creating a single central channel through which the buffer volume flow was controlled via a syringe pump (NE‐1000, New Era Pump Systems). Parafilm gaskets as well as coverslips with buffer inlets and additional apertures for temperature sensors (see Figure S2) were prepared with a laser engraver (VLS2.30, Universal Laser Systems).

*Temperature Management Assembly*: The thermal control system was developed following an approach originally proposed to reduce laser‐induced drift in optical tweezers.^28^ Polyimide foil heaters (Minco) were attached to the barrel of the objective (Heater 1: resistance *R*_1_ = 33.9 Ω) and the bottom of the sample holder baseplate (Heaters 2a/b: resistance *R*_2_ = *R*_2a_ + *R*_2b_ = 2 × 5.6 Ω = 11.2 Ω) with silicone stretch tape (Minco) and pressure‐sensitive adhesive (Minco), respectively. The heating foils were connected to the voltage outputs of two programmable power supply units (TTi; resolution: 1 mV) controlled by a computer via USB. Resistive platinum Pt100 temperature detectors (Correge; precision: ±0.04 Ω ↔ ±0.01 °C) were fixed to objective and baseplate via thermally conductive double‐sided adhesive tape (Thorlabs) and thermally conductive epoxy (Minco), respectively. To minimize errors due to lead resistances, each Pt100 sensor was connected in a three‐wire configuration^44^ to a temperature converter (Brodersen; voltage range: 0–10 V) providing a signal of the following type: *V*_conv_ = (*T* + 50 °C)/15 °C·V^−1^. We adjusted the converter gains such that the system returned the desired voltages at two reference temperatures. The signals were fed into an external data acquisition (DAQ) module (National Instruments) connected to the computer by USB. Table S2 contains the exact references of all relevant components used, valid for both MT configurations.

*Proportional‐Integral‐Derivative (PID) Feedback*: Temperature data were acquired and processed, and heating foil voltages updated at a frequency of 2 Hz by means of customized software developed in LabVIEW (National Instruments). The application contains two standard PID controllers working in parallel, one for the objective and another for the baseplate heating circuit. We applied the Callendar–Van Dusen equation^44^ to the acquired temperatures to correct for nonlinearities in the *R*(*T*)‐response of the Pt100 thermometers. The sensitivities *S* (in V^2^·°C^−1^) for calculating the initial voltage setpoints delivered to the power supplies were determined offline by independently raising the voltage of each heater circuit stepwise and annotating the mean equilibrated temperature at least 40 minutes after each step (see Figure 2A). Lowering the voltage in the same way afterwards gave rise to equivalent temperature values (data not shown). The conversion factors *S*’ (in V·°C^−1^) actually used to quantify the voltage corrections during continuous PID feedback were optimized empirically online. The P, I and D gains were tuned according to the Ziegler–Nichols step response (open‐loop) and frequency response (closed‐loop) methods.^45^ Typical feedback parameters yielding system performances as depicted in Figure 2B can be found for MT_1_ in Table S3; parameters for MT_2_ were similar (data not shown).

*Buffer Temperature Calibration Experiments*: For all calibrations the sample stage was centered in XY as represented in Figure 1. We located auxiliary Parafilm pieces with rectangular apertures of the size of the Pt100 temperature sensors onto a fluid chamber with extra openings of slightly larger dimensions near channel center and inlet (see Figure S2C/D). With the help of some vacuum grease we could thus establish a leak‐less seal for thermometers T3 and T4 to probe the temperature inside the chamber without touching the lower coverslip. The ambient temperature during all measurements lay in the range of 23–26 °C. To determine the heat transduction efficiencies between objective (baseplate) and sample cell center (edges), equal pairs of at least four increasing setpoints between 27 and 39 °C were applied to the two heating circuits and sensors T3 and T4 monitored with the buffer at rest (see Figure 3A). Decreasing the setpoints afterwards resulted in similar temperature readings (data not shown). Subsequently, the corrected setpoints yielding equivalent thermal conditions near center and inlet (see Results) were checked for accuracy within a range of 25–41 °C by collecting data from several fluid chambers under different buffer flow conditions (see Figure 3B). The calibration holds equally well for both MT configurations.

*MT Single‐Molecule Translocation Assays*: In experiments with AddAB, after having set the desired temperature inside the sample cell, the biotinylated enzyme – previously bound to the near end of the dsDNA substrate – was tethered between (i) a 1 μm streptavidin‐coated superparamagnetic microsphere (MyOne Dynabeads, Invitrogen) and (ii) the lower fluid chamber surface via a single digoxigenin–anti‐digoxigenin interaction at the far DNA end. A second identical microsphere unspecifically attached to the chamber surface acted as a reference for inferring Z‐positions of AddAB from microsphere height differences^46^ (see Figure 4A) at a constant camera frame rate of 60 Hz in both MT configurations (spatial resolution ∼5–10 nm at 3 pN of force). For all MT measurements, the reaction buffer included tris acetate (25 mm, pH 7.5), magnesium acetate (2 mm), and dithiothreitol (DTT; 1 mm); ATP (1 or 4 mm) was added to start AddAB activity. The assays shown herein were carried out in a constant flow of (preheated) buffer at 65 μL·min^−1^ ≈ 1 μL·s^−1^ (a compromise ensuring reliable video tracking at sufficiently short reaction onset times) and with a pulling force between 3 and 14 pN; their corresponding data were treated as explained before.^18^ Briefly, instantaneous and mean translocation velocities were determined from derivatives of the position‐vs.‐time signals previously smoothed from 60 to 3 Hz – the latter without taking into account the early ATP gradient and random pauses (if any) of the enzyme along its track. To obtain the histograms of instantaneous rates v*_MT_ shown in Figure 6, the interval ∼2001–4000 bp from the proximal end of the Chi10‐Rev‐Rev substrate was chosen because it provided maximum statistics within a region after the initial ATP concentration rise and before any individual Chi sequences (see Figure 4B). In all translocation traces, we converted the raw distance data given in micrometers to (kilo)base pairs of DNA through division by the rise‐per‐base‐pair value defined by the inextensible worm‐like chain model^47^ for a given applied force. Taking into account the temperature dependences of fractional extension *z*/*L*_0_ (*z* being the end‐to‐end distance and *L*_0_ the contour length of the molecule) and persistence length *L*_p_,^48^ we used the following parameters (in nm·bp^−1^): at 3 pN, 0.3138 (24–25 °C), 0.3132 (27–28 °C), 0.3123 (30 °C), 0.3113 (33 °C), and 0.3097 (37 °C); at 8 pN, 0.3237 (24–25 °C) and 0.3221 (30 °C); at 12 pN, 0.3268 (24–25 °C); and at 14 pN, 0.3278 (24–25 °C).

*Triplex Displacement Ensemble Measurements*: TFO displacement assays were performed in a stopped‐flow fluorimeter (SF‐61DX2, TgK Scientific) using slight modifications of published methods;^39^, ^49^ in particular, the data presented herein were obtained with the distal end of the DNA substrate left unblocked (see Figure S4). The stated temperatures correspond to the values returned from a circulating water bath that controls the fluorimeter and yields an estimated accuracy of ±0.1 °C (see supplementary data of Reference 13). All concentrations quoted below are final, after mixing in the stopped‐flow apparatus. The TAMRA‐labelled triplex – located with its far end at 924 bp from the proximal DNA end – was excited at 547 nm with the slits set at 5.4 nm and the fluorescence monitored above 570 nm. AddAB (5 nm) was pre‐bound to substrate DNA (0.2 nm) in a buffer containing tris acetate (25 mm, pH 7.5), magnesium acetate (2 mm), DTT (1 mm) and BSA (100 μg·mL^−1^; Sigma). Rapid mixing against a solution of the same buffer containing ATP (1 mm) initiated translocation, and the fluorescence changes resulting from TFO dissociation were recorded. The data were fitted using GraphPad Prism to a single exponential that was offset on the X‐axis to define a lag time *t*_1_ before TFO displacement (see Figure S4). This value is taken as the sum of the time constants for all processes that result in the arrival of the enzyme at the triplex (see Reference 49 for discussion and further details about the method). As translocation initiation events are very brief for AddAB,[[qv: 19d]],^19^, ^39^, the distance travelled divided by the lag time yields an approximate translocation rate for any given temperature, as shown in Figure 5.

## Supporting information

As a service to our authors and readers, this journal provides supporting information supplied by the authors. Such materials are peer reviewed and may be re‐organized for online delivery, but are not copy‐edited or typeset. Technical support issues arising from supporting information (other than missing files) should be addressed to the authors.

Supplementary
