# Supplementary material for: Probing DNA Helicase Kinetics with Temperature‐Controlled Magnetic Tweezers
Source: Small. 2014 Nov 14;11(11):1273–84. doi: 10.1002/smll.201402686 (PMC4473356; doi:10.1002/smll.201402686)
Supplement: Supplementary file 1 — Supplementary [file SMLL-11-1273-s001.pdf]

Copyright WILEY-VCH Verlag GmbH & Co. KGaA, 69469 Weinheim, Germany, 2014.

Supporting Information

for *Small,* DOI: 10.1002/smll.201402686

Probing DNA Helicase Kinetics with Temperature-Controlled Magnetic Tweezers

*Benjamin Gollnick, Carolina Carrasco, Francesca Zuttion, Neville S. Gilhooly, Mark S. Dillingham, and Fernando Moreno-Herrero**

Copyright WILEY-VCH Verlag GmbH & Co. KGaA, 69469 Weinheim, Germany, 2014.

Supporting Information

Probing DNA Helicase Kinetics with Temperature-Controlled Magnetic Tweezers

*Benjamin Gollnick, Carolina Carrasco, Francesca Zuttion, Neville S. Gilhooly, Mark S. Dillingham, and Fernando Moreno-Herrero**

SUPPLEMENTARY EXPERIMENTAL SECTION

Estimates of linear flow velocities through magnetic tweezers (MT) sample cells

The uniform laminar flow through a practically infinite channel of length l and rectangular cross section ($d \times w$, where d and w are channel height and width, respectively) can be approximated by the same type of flow through a circular tube (see e.g. Reference [1]) with hydraulic radius $r = (d \times w)/(d + w)$; in our case: $l \approx 25$ mm for $w \approx 7$ mm (see Figure S2C), and $d \approx 200$ (100) µm for thick (thin) fluid chambers. The linear velocity profile far from the entrance can then be expressed as

$$v_{\mathrm{flow}}(z) = v_{\max} \times \left(1 - \frac{(r-z)^2}{r^2}\right) \quad ,$$

with z being the distance from the boundary and $v_{\max} = 2 \times v_{\mathrm{mean}}$ the maximum flow at $z = r$, just twice the average value (see e.g. Reference [1]). MT experiments ($z \ll d \sim r$, see Figure 4A) always correspond to the case $v_{\mathrm{flow}}(z) \ll v_{\max}$. Choosing $z = 2$ µm (roughly the initial distance of the AddAB protein from the coverslip, see Figure 4B), the velocities in X for our thick ($r \approx 190$ µm) and thin ($r \approx 100$ µm) cells are $v_{\mathrm{flow\_thick}}(2\ \mu\mathrm{m}) \approx 0.02 \times v_{\mathrm{max\_thick}}$ and $v_{\mathrm{flow\_thin}}(2\ \mu\mathrm{m}) \approx 0.04 \times v_{\mathrm{max\_thin}}$. Considering that the average stationary flow depends inversely on the cross-sectional area of the channel, our rectangular chamber yields $v_{\mathrm{max\_thin}} = 2 \times v_{\mathrm{max\_thick}}$ and therefore $v_{\mathrm{flow\_thin}}(2\ \mu\mathrm{m})/v_{\mathrm{flow\_thick}}(2\ \mu\mathrm{m}) \approx 4$. Note that in spite of decreasing absolute values, this flow ratio stays practically constant as the enzyme translocates towards the glass surface, i.e. for $z \to 0$. The linear flow velocities for a constant volume flux of ~1 $\mu\mathrm{L}\cdot\mathrm{s}^{-1}$ read as follows: $v_{\mathrm{max\_thick}} \approx 1.43\ \mathrm{mm}\cdot\mathrm{s}^{-1}$, $v_{\mathrm{max\_thin}} \approx 2.86\ \mathrm{mm}\cdot\mathrm{s}^{-1}$; $v_{\mathrm{flow\_thick}}(2\ \mu\mathrm{m}) \approx 30\ \mu\mathrm{m}\cdot\mathrm{s}^{-1}$, $v_{\mathrm{flow\_thin}}(2\ \mu\mathrm{m}) \approx 110\ \mu\mathrm{m}\cdot\mathrm{s}^{-1}$. Assuming for instance an initial adenosine triphosphate (ATP) gradient region on the order of 1 mm along the X-axis, these flow estimates imply that a maximum ATP concentration at $z \leq 2$ µm is reached after at least 33 (9) seconds of flushing buffer through thick (thin) fluid chambers, which should be longer than the total translocation time of AddAB in the first case (see Figure 4B and Discussion in the main manuscript).

Uncertainty propagation for activation energy E_a from single-molecule experiments

Rearranging the Arrhenius relationship (Equation 1 of the main text) gives the following expression for the activation energy barrier of translocation: $E_a = k_B T \times [\ln(v_0) - \ln(v(T))]$, where ln denotes the natural logarithm. Assuming error bars arising from normally distributed data in Figure 5, we can estimate the uncertainty of $E_a(v(T),T)$ induced by the uncertainties of the measured variables $v(T)$ (velocity) and T (temperature) using the Gaussian propagation formalism (see e.g. Reference [2]):

$$u(E_a) = \sqrt{\left(\frac{\partial E_a}{\partial v(T)} \times u(v(T))\right)^2 + \left(\frac{\partial E_a}{\partial T} \times u(T)\right)^2 + 2\times\left(\frac{\partial E_a}{\partial v(T)} \times \frac{\partial E_a}{\partial T} \times \operatorname{cov}(v(T),T)\right)}$$
$$= \sqrt{\left(-\frac{u(v(T))}{v(T)} k_B T\right)^2 + \left(\frac{u(T)}{T} E_a\right)^2 - 2\times\left(\frac{u(v(T))}{v(T)} k_B T \times \frac{u(T)}{T} E_a \times \rho(v(T),T)\right)} \quad .$$

Here, $u(x)$ indicates the uncertainty of the corresponding argument x, $(\partial E_a / \partial x)$ the partial derivative of E_a with respect to x, and $\operatorname{cov}(x,y)$ the covariance of x and y. To assess an upper limit of $u(E_a)$, we can neglect the third summand under the second square root – which contains the correlation coefficient $\rho(v(T),T) \equiv \operatorname{cov}(v(T),T) / [u(v(T)) \times u(T)]$, with $0 < \rho < 1$ since $v(T)$ increases with T – because its contribution is negative. This yields

$$u(E_a) \leq \sqrt{\left(\frac{u(v(T))}{v(T)} k_B T\right)^2 + \left(\frac{u(T)}{T} E_a\right)^2}$$
$$\approx \sqrt{\left(\left\langle\frac{u(v(T))}{v(T)}\right\rangle k_B T\right)^2 + \left(\left\langle\frac{u(T)}{T}\right\rangle E_a\right)^2} \quad ,$$

where the five translocation rate and temperature mean values, standard deviations (SDs, in case of $v(T)$) and accuracies (in case of T) corresponding to initial MT experiments (filled squares in Figure 5) at 3 piconewtons (pN) allow us to evaluate mean relative errors $\langle u(v(T)) / v(T)\rangle \approx 15.1$ % and $\langle u(T) / T \rangle \approx 0.2$ % that can serve as approximations of the exact values. Inserting them into the final expression above and setting $E_a = 21.4\ k_B T$ gives $u(E_a) \leq 0.16\ k_B T \approx 0.2\ k_B T$.

References

[1] Y. A. Çengel, J. M. Cimbala, *Fluid Mechanics: Fundamentals and Applications*, 1st ed., McGraw-Hill, New York, NY, USA **2006**.

[2] J. R. Taylor, *An Introduction to Error Analysis: The Study of Uncertainties in Physical Measurements*, 2nd ed., University Science Books, Sausalito, CA, USA **1997**.

SUPPLEMENTARY TECHNICAL INFORMATION

Table S1. Principal differences between both magnetic tweezers (MT) configurations.

Component	MT_1	MT_2
Cubic magnets (edge length = 5 mm)	2 × W-05-G (Supermagnete); horizontal alignment (gap size ≥ 2 mm)	2 × W-05-N50-G (Supermagnete); vertical alignment (gap size ≈ 0.2 mm)
Oil-immersion objective	PLAPON 60XO (Olympus)	UPLSAPO 100XO (Olympus)
Video camera	CCD[a)]; TM-6710CL (JAI Pulnix); max. frame rate at full sensor readout: 120 Hz	CMOS[b)]; MC1362 (Mikrotron); max. frame rate at full sensor readout: 500 Hz

[a)] CCD = charge-coupled device; [b)] CMOS = complementary metal–oxide semiconductor.

Table S2. References to the main commercial components of the temperature control assembly.

Description	Manufacturer	Count	Reference	Comments
Polyimide heater (Heater 1)	Minco	1	HK5210R33.9L12A	Without adhesive backing
Polyimide heater (Heaters 2a/b)	Minco	2	HK5160R5.6L12E	Backed with pressure-sensitive adhesive
Pt100 temperature sensor (T1–T4)	Correge	4	PTFC101T	Probe size ($W \times L$): 2.0 × 2.3 mm
Temperature signal converter	Brodersen	4	PXT-10.924	One for each sensor
Data acquisition module (with chassis)	National Instruments (NI)	1	NI 9215 BNC (with cDAQ-9171)	Provides up to four readouts in parallel
Programmable power supply unit	Thurlby Thandar Instruments (TTi)	2	PL303-P	One per heating circuit
Fixed power supply unit	ELC	1	AL912A	Powers the signal converters
Silicone stretch tape	Minco	1	#20 stretch tape	For fixing Heater 1 to the objective
Thermally conductive double-sided tape	Thorlabs	1	TCDT1	For fixing sensor T1 to the objective
Thermally conductive epoxy glue	Minco	1	#15 epoxy	For fixing sensor T2 to the baseplate

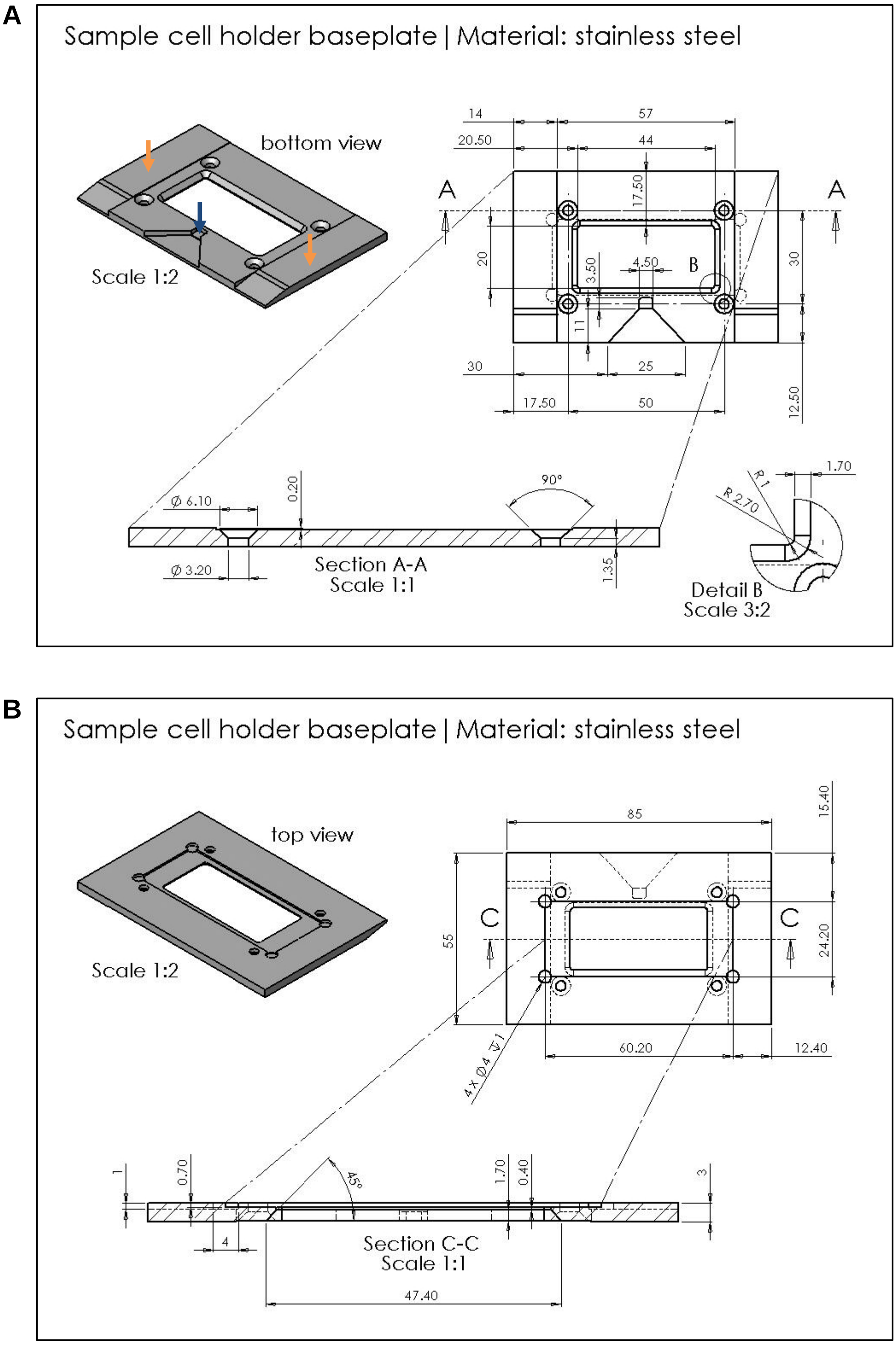

Figure S1. Technical drawings of the sample cell holder baseplate. All dimensions are in millimeters. (**A**) Bottom view. The areas for attachment of Heaters 2a/b and temperature sensor T2 are indicated by orange and dark blue arrows, respectively. Compare also with Figure S2B. (**B**) Top view. The central indentation accepts a sample chamber of outer dimensions 60 × 24 mm in XY with a maximum thickness of 0.7 mm.

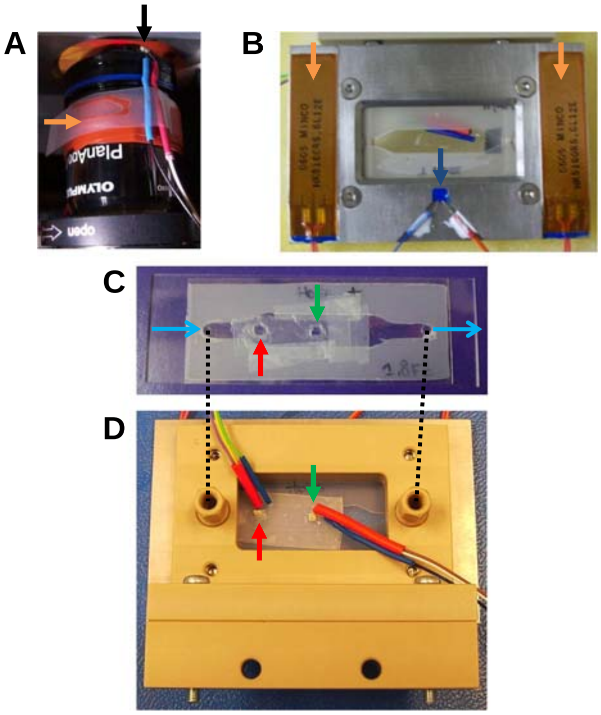

Figure S2. Optical images (not to scale) of crucial elements of the temperature control system according to Figure 1. (**A**) Side view of the objective heating assembly. Arrows indicate Heater 1 (orange, covered by red stretch tape) and sensor T1 (black, attached close to the immersion oil drop). (**B**) Bottom view of the baseplate heating assembly. Arrows indicate Heaters 2a/b (orange) and sensor T2 (dark blue, buried in blue epoxy). For the exact dimensions, compare with Figure S1A. (**C**) Fluid chamber with additional apertures to calibrate the buffer temperature near center (green arrow) and inlet (red arrow). Plastic paraffin film (Parafilm) pieces for sealing are visible on the top coverslip. The flow direction is indicated by light blue arrows. (**D**) The complete sample cell assembly ready for temperature calibration. The fluid chamber from (C) now sits between the baseplate and a top part made of polyether ether ketone (PEEK). Arrows indicate sensor T3 (green) and T4 (red), both contacting the buffer and sealed from the outside with Parafilm and vacuum grease.

Table S3. Common <u>P</u>roportional-<u>I</u>ntegral-<u>D</u>erivative feedback control parameters for microscope objective and baseplate heating circuits.

Heater circuit	Sensitivity S (V^2·°C^{-1})[a)]	Conversion factor S' (V·°C^{-1})[b)]	P gain	I gain	D gain
1 (objective)	4.3	0.33	0.48	2.0×10^{-3}	26
2 (baseplate)	1.0	0.20	0.05	0.1×10^{-3}	8

[a)] for initial voltage setpoint calculations (see Figure 2A); [b)] for fast voltage corrections (yields best PID controller performance).

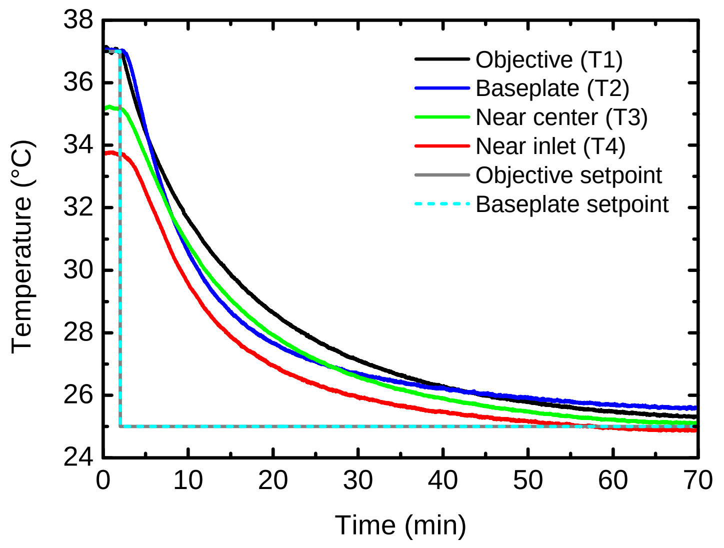

Figure S3. Time course of the typical cooling behavior during calibration measurements. With the previous setpoints at 37 °C, after two minutes both heating circuits are shut off. Subsequently, the system needs typically one hour to equilibrate (ambient temperature ~25 °C). Initial relaxation of the temperatures inside the sample chamber (during the first ~10 min) takes place at an approximate rate of −0.5 $°C \cdot min^{-1}$.

Comments on heating effectiveness and feedback response times

The initial calibration of sensitivities S (see Figure 2A and Table S3) resulted in the Heater 1 circuit having a roughly four-times higher S-value than Heaters 2a/b connected in series, with a resistance ratio of $R_1 / R_2 = 33.9\ \Omega / 11.2\ \Omega \approx 3$. Neglecting detrimental effects such as self-heating, this leads to effective heat powers per unit temperature of $p_{heat\_1} = S_1 / R_1 \approx 130$ $mW \cdot °C^{-1}$ and $p_{heat\_2} = S_2 / R_2 \approx 90$ $mW \cdot °C^{-1}$, respectively, showing that in our configuration baseplate heating is slightly more efficient than objective heating in terms of the dissipated energy per time.

The low proportional/integral (P/I) and high derivative (D) gains (see Table S3) obtained during feedback parameter optimization are indicative of slow processes and necessary to compensate significant delays between the temperatures observed at heat source and sensing thermometer. This is particularly true for the baseplate circuit, whose temperature sensor T2 is located ~3 cm away from the heating foils Heaters 2a/b (see Figure S1A) and which consequently shows pronounced signal overshoots after a setpoint change. The absence of an active cooling mechanism requires additional waiting times when the temperature setpoints are lowered considerably (see Figure S3).

SUPPLEMENTARY DATA OF TRANSLOCATION RATE MEASUREMENTS

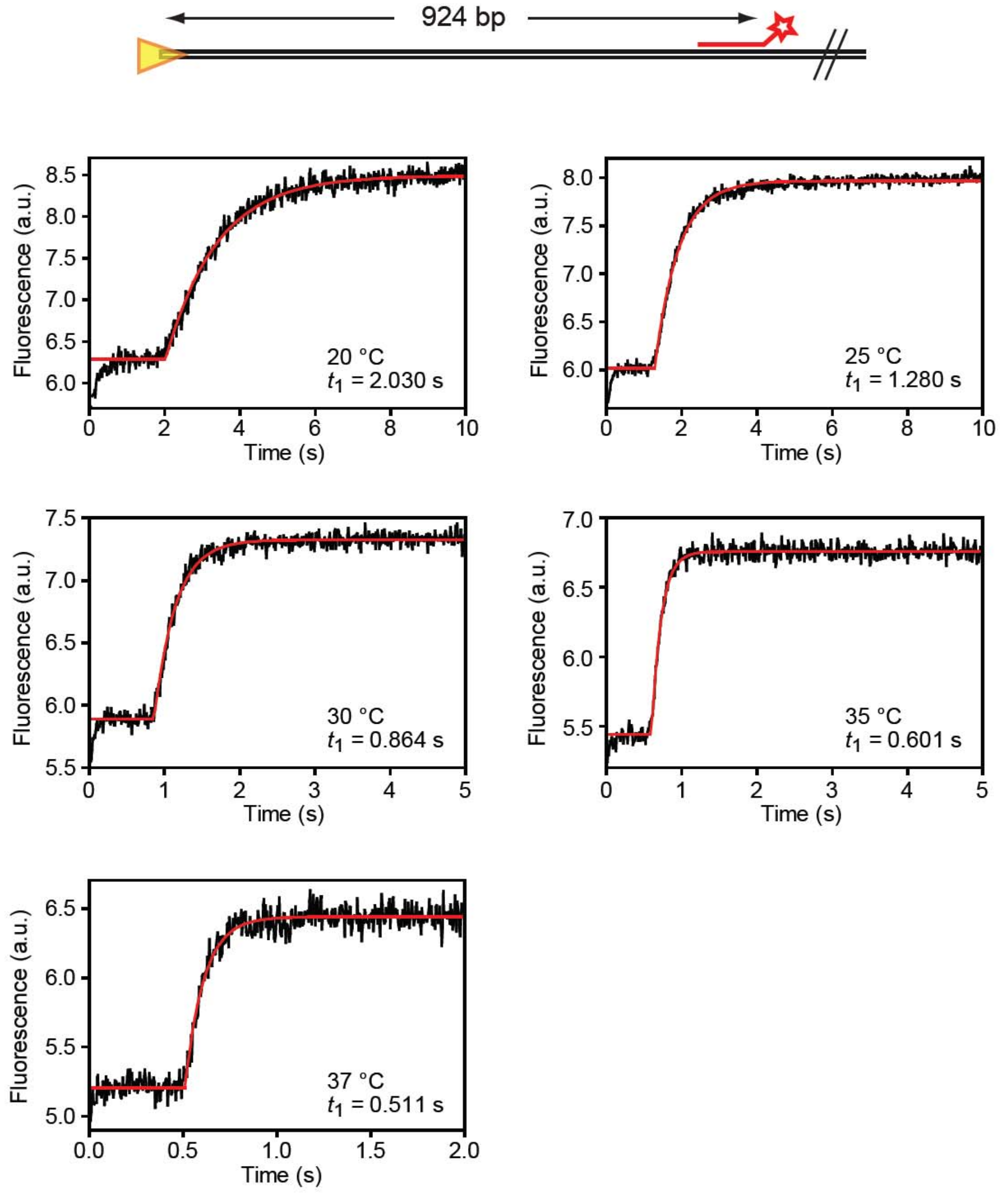

Figure S4. Experimental configuration and example traces of stopped-flow triplex displacement assays at all studied temperatures. A large population of pre-bound AddAB molecules – ideally just one protein (yellow triangle) per DNA substrate – starts to translocate synchronously at time $t_0 \approx 0$ towards a tetramethylrhodamine (TAMRA)-labelled triplex-forming oligonucleotide (TFO, red handle with star-shaped end). TFO displacement causes an increase in the detected fluorescence intensity. The lag time t_1, which is determined from offset exponential fits (red curves) of averaged stopped-flow traces (black data points), corresponds to the time needed for AddAB to reach the triplex and decreases with increasing temperature. Dividing the distance – 924 base pairs (bp) – of the TFO binding site from the proximal DNA end by the different t_1-values yields the apparent translocation velocities plotted in Figure 5.

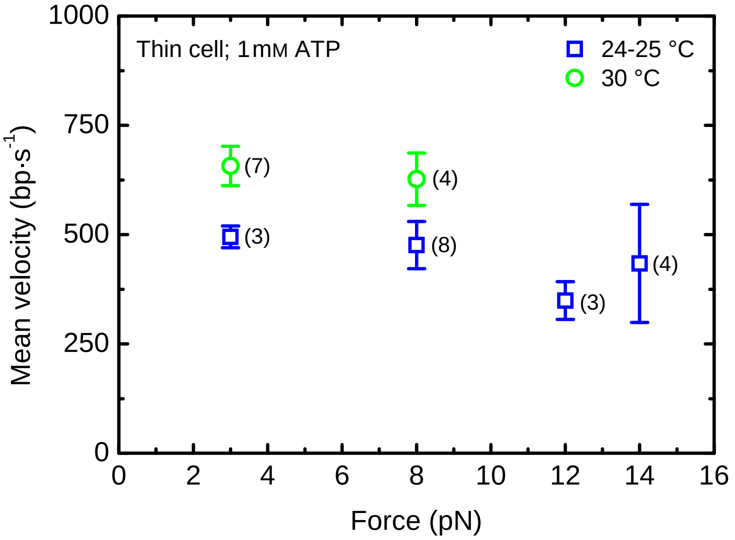

Figure S5. Force dependence of single-molecule translocation rates. Using the thermally stabilized magnetic tweezers (MT) instrument in configuration MT_2 (see Table S1) with thin (100 µm) sample cells and reaction buffer containing 1 mM of adenosine triphosphate (ATP; see Experimental Section), the mean translocation velocity of AddAB is probed at loads (F_Z in Figure 4A) between 3 and 14 piconewtons (pN) and two different temperatures. The digit next to each data point indicates the number of DNA molecules used for calculating the average. Error bars represent the standard deviation (SD), the typical relative error in F_Z (no error bars shown) being 5–10 %. For both temperature settings, no significant rate change – at most a subtle decrease at ambient conditions above 8 pN – occurs within the studied force range.
